# Phagocytosis by macrophages depends on histamine H2 receptor signaling and scavenger receptor 1

**DOI:** 10.1002/mbo3.908

**Published:** 2019-08-01

**Authors:** Robert Fultz, Melinda A. Engevik, Zhongcheng Shi, Anne Hall, Beatrice Herrmann, Bhanu P. Ganesh, Angela Major, Anthony Haag, Yuko Mori‐Akiyama, James Versalovic

**Affiliations:** ^1^ Department of Pathology Texas Children’s Hospital Houston TX USA; ^2^ Integrative Program in Molecular and Biomedical Sciences Baylor College of Medicine Houston TX USA; ^3^ Department of Pathology & Immunology Baylor College of Medicine Houston TX USA; ^4^ Molecular Virology & Microbiology Baylor College of Medicine Houston TX USA; ^5^ Cell and Molecular Biology Graduate Group University of Pennsylvania Philadelphia PA USA; ^6^ Department of Neurology University of Texas Health Science Center at Houston Houston TX USA

**Keywords:** Atg12, autophagy, bacteria, Beclin‐1, Hrh2, innate immunity, Msr1

## Abstract

The histamine H2 receptor (H2R) is a G protein‐coupled receptor that mediates cyclic AMP production, protein kinase A activation, and MAP kinase signaling. In order to explore the multifaceted effects of histamine signaling on immune cells, phagocytosis was evaluated using primary mouse‐derived macrophages. Phagocytosis is initiated by signaling via surface‐bound scavenger receptors and can be regulated by autophagy. Absence of H2R signaling resulted in diminished phagocytosis of live bacteria and synthetic microspheres by primary macrophages from histamine H2 receptor gene (*Hrh2*)‐deficient mice. Flow cytometry and immunofluorescence microscopy were used to quantify phagocytosis of phylogenetically diverse bacteria as well as microspheres of defined chemical composition. Autophagy and scavenger receptor gene expression were quantified in macrophages after exposure to *Escherichia coli*. Expression of the autophagy genes, *Becn1* and *Atg12,* was increased in *Hrh2* ^−/−^ macrophages, indicating upregulation of autophagy pathways. Expression of the Macrophage Scavenger Receptor 1 gene (*Msr1*) was diminished in *Hrh2*‐deficient macrophages, supporting the possible importance of histamine signaling in scavenger receptor abundance and macrophage function. Flow cytometry confirmed diminished MSR1 surface abundance in *Hrh2* ^−/−^ macrophages. These data suggest that H2R signaling is required for effective phagocytosis by regulating the process of autophagy and scavenger receptor MSR1 abundance in macrophages.

## Graphical abstract

We demonstrate that histamine 2 receptor is required for phagocytosis of bacteria and microspheres by mouse macrophages. Expression of key autophagy pathway genes is increased, and Macrophage Scavenger Receptor 1 is decreased in abundance in histamine 2 receptor‐deficient macrophages.

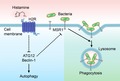

## 1 INTRODUCTION

Macrophages are prominent components of the innate immune system and contribute to the functional integrity of the intestinal mucosa. These cells play central roles in immune surveillance, phagocytosis, antigen presentation, tissue development, wound healing, and overall intestinal homeostasis. Macrophage‐mediated phagocytosis serves as a front line of defense and is critical for eliminating invading pathogens (Elliott & Ravichandran, 2010; Levin, Grinstein, & Canton, 2016; Levin et al., 2017). Patients with defects in phagocytosis are prone to earlier dissemination of infection, severe sepsis, and subsequently suffer increased mortality (Andrews & Sullivan, 2003; Kim et al., 2017). Reduced phagocytic activity during the first 24 hr after hospital admission is also a known negative predictor for survival in septic patients (Danikas, Karakantza, Theodorou, Sakellaropoulos, & Gogos, 2008). In patients with chronic obstructive pulmonary disease, reduced phagocytosis of common airway pathogens enables bacterial persistence in the lower airways and may contribute to chronic inflammation (Taylor et al., 2010). Given the importance of macrophages in health and disease, elucidating molecular mechanisms that regulate macrophage function and particularly phagocytosis are important for understanding innate immunity.

Phagocytosis is a complex immune process that results in removal and elimination of infectious agents including bacterial cells, misplaced microbes, apoptotic cells, neoplastic cells, or cellular debris. In general, bacterial antigens are recognized via Toll‐like receptors (TLRs), pathogen‐associated molecular patterns (PAMPs), and NOD‐like receptors (NLRs) (Hisamatsu, Ogata, & Hibi, 2008; Rioux et al., 2007; Sheikh & Plevy, 2010). Phagocytosis is commonly initiated by the engagement of surface receptors that trigger plasma membrane and actin cytoskeleton remodeling (Levin et al., 2016, 2017). This process produces pseudopods that gradually enlarge and engulf the target. Engulfed microparticles enter the phagosome and are subsequently digested. Phagocytosis of microbes differs in terms of relative efficiency among different microbial species, and some organisms may be able to evade or escape killing entirely by professional phagocytes (Perun et al., 2017). Recently, autophagy and phagolysosomal function have also emerged as fundamental elements involved in phagocytosis of invasive microbes (Bonilla et al., 2013; Lima et al., 2011; Rioux et al., 2007). Activation of autophagy has been shown to suppress phagocytosis (Lima et al., 2011; Martinet, Schrijvers, Timmermans, Herman, & De Meyer, 2009), while inhibition or loss of autophagy has been shown to enhance phagocytosis (Bonilla et al., 2013; Song et al., 2017; Zhu, Li, Ding, & Wang, 2018). Importantly, this relationship is mediated by transcriptional control of scavenger receptors by autophagy‐related gene products (Bonilla et al., 2013).

Numerous factors have been shown to influence macrophage phagocytosis (Aderem & Underhill, 1999; Anand et al., 2007; Bonilla et al., 2013; Das et al., 2015; van Ham, Kokel, & Peterson, 2012; He et al., 2018; Kedzierska et al., 2003; Kim et al., 2017; Li et al., 2005; Martinet et al., 2009; Perun et al., 2017; Shiratsuchi & Basson, 2004; Song et al., 2017; Wallace et al., 2018), including histamine signaling. Macrophages express four histamine receptors (H1R, H2R, H3R, and H4R), and the type of receptor activated dictates the cellular response. In immune cells, histamine H1 receptor (H1R) is classically considered to be pro‐inflammatory and cell‐activating, while H2R is considered tolerogenic and anti‐inflammatory (O'Mahony, Akdis, & Akdis, 2011). In contrast, other reports found that histamine activation of H4R induced macrophage chemotaxis and phagocytosis in murine RAW264.7 and bone marrow‐derived macrophages (BMMs) (Czerner, Klos, Seifert, & Neumann, 2014). In this same study, inhibition of H1R with mepyramine or inhibition of H2R with famotidine had no effect on chemotaxis or phagocytosis (Czerner et al., 2014). In human alveolar macrophages, addition of picomolar concentrations of histamine acted via the H2R to promote chemotaxis (Radermecker, Bury, & Saint‐Remy, 1989). In this model, inhibition of H1R with promethazine had no effect. These studies have exclusively used pharmacological inhibitors to block target histamine receptors, which are known to have some degree of off‐target binding. Therefore, to circumvent issues arising from the use of pharmacological inhibitors, we have used the CRISPR‐Cas9 system to genetically modify C57BL/6J mice, by deletion of functional exons, generating mouse models with cells lacking *Hrh1* or *Hrh2*.

Using macrophages derived from histamine receptor‐deficient mice, we demonstrated that loss of *Hrh2*, but not *Hrh1*, results in decreased phagocytosis efficiency in both bone marrow‐derived and peritoneal macrophages. Phagocytosis of phylogenetically diverse bacteria as well as synthetic microspheres is reduced. We determined that Macrophage Scavenger Receptor 1 surface abundance is diminished in *Hrh2* ^−/−^ bone marrow‐derived macrophages which accounts for reduced phagocytosis. Additionally, we demonstrate that expression of key autophagy genes, *Atg12* and *Becn1,* is increased which may be responsible for suppression of scavenger receptor expression.

## 2 RESULTS

### 2.1 *Hrh2* is required for phagocytosis by bone marrow‐derived macrophages (BMMs)

**Figure A1:**
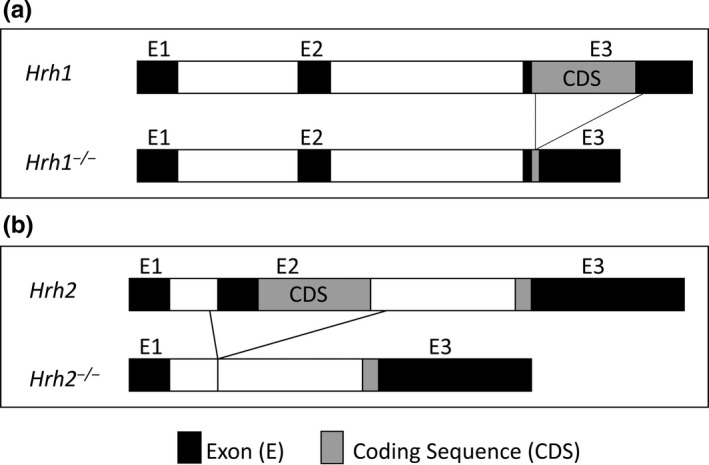
Development of *Hrh1*‐ and *Hrh2*‐deficient mouse models. C57B6 mice *Hrh1*‐ and *Hrh2*‐knockout mice were generated by CRISPR‐Cas9‐mediated genome editing of functional exons

*Hrh1*‐deficient and *Hrh2*‐deficient mice were generated by CRISPR‐Cas9‐mediated genome editing. Sequencing confirmed that a 1,546 bp fragment of the *Hrh1* gene and a 1,960 bp fragment of the *Hrh2* gene were deleted in each mouse (Figure A1 in Appendix). Lack of expression was verified by qPCR of isolated macrophages. These mice are still capable of producing histamine by decarboxylation of L‐histidine, and, aside from endogenous histamine produced by mammalian cells, epithelial and immune cells of the intestinal mucosa may be exposed to histamine generated by microbial cells in the intestine (Barcik et al., 2016).

**Figure 1:**
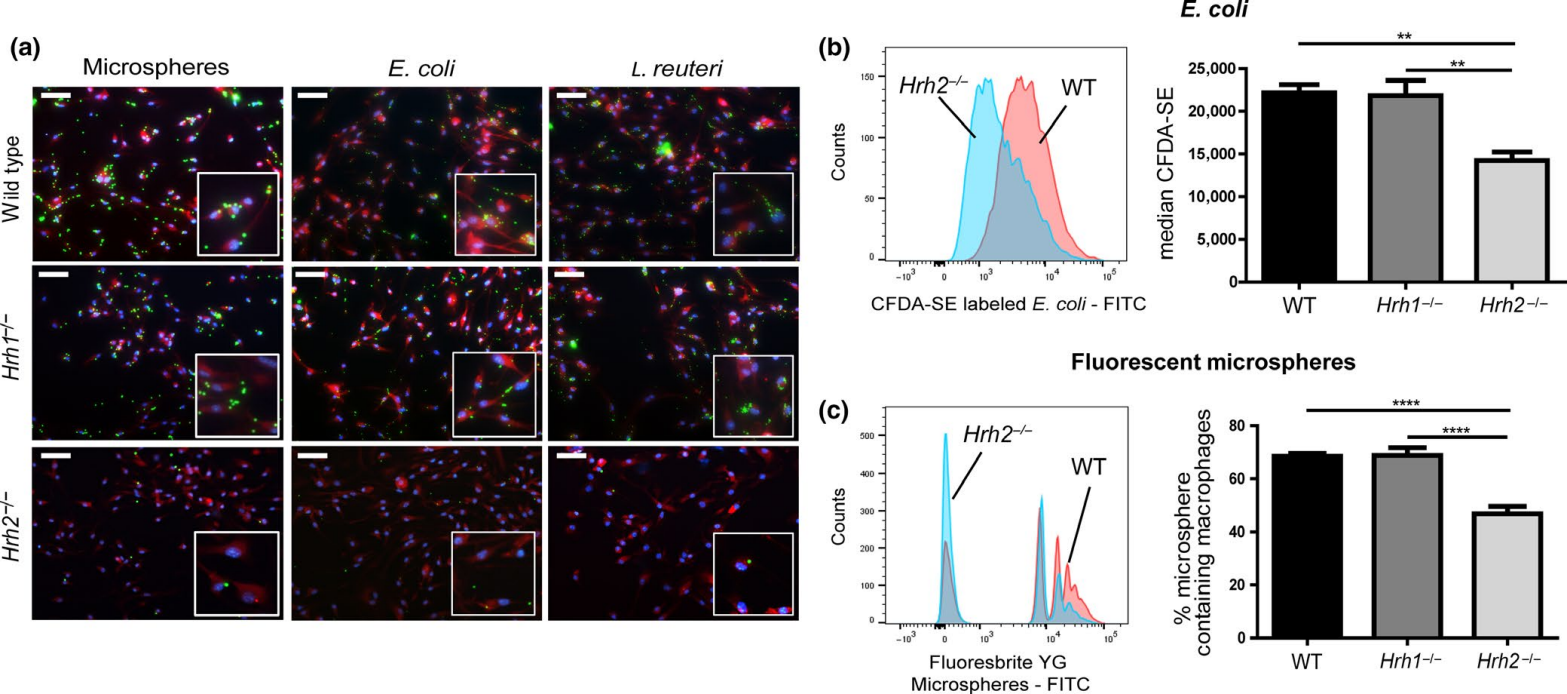
Phagocytosis is reduced in *Hrh2* ^−/−^ bone marrow‐derived macrophages. Phagocytosis of fluorescently labeled bacteria and microspheres was observed in WT, *Hrh1* ^−/−^, and *Hrh2* ^−/−^ bone marrow‐derived macrophages. (a) Fluorescence microscopy showed reduced phagocytosis of Fluoresbrite YG microspheres (2 µm) and CFDA‐SE‐labeled *Escherichia coli* and *Lactobacillus reuteri* (green) by *Hrh2* ^−/−^ macrophages compared to WT or *Hrh1* ^−/−^ controls. Cells were counterstained with Phalloidin (red), and nuclei were dyed with Hoechst (blue). Bars represent 50 µm, and inset boxes are expanded views of fields of interest. (b and c) Flow cytometry analysis of bone marrow‐derived macrophages exposed to fluorescent microspheres or CFDA‐SE‐labeled bacteria confirmed reduced phagocytosis of fluorescent microparticles by *Hrh2* ^−/−^ bone marrow‐derived macrophages. Cells were stained with viability stain, and upon analysis, fluorescence intensity in the FITC channel was measured and compared among populations. Statistical analysis was performed by two‐way ANOVA of mean with Bonferroni multiple comparisons. **p < .01, ****p < .0001; *n* = 3

To characterize phagocytosis by *Hrh1* ^−/−^ and *Hrh2* ^−/−^ murine macrophages, BMMs were exposed to CFDA‐SE‐labeled *Escherichia coli* K‐12, CFDA‐SE‐labeled *Lactobacillus reuteri* ATCC PTA 6475, or Fluoresbrite YG microspheres (2 µm in diameter). CFDA‐SE‐labeled cells and microspheres labeled with Fluoresbrite YG yield peak fluorescence emission signals at 517 nm and 485 nm, respectively, near the peak FITC fluorescence emission wavelength, which is 519 nm. Fluorescence microscopy of macrophages exposed to fluorescent bacteria or microspheres (Figure 1a) shows diminished phagocytosis by *Hrh2* ^−/−^ BMMs. Additionally, BMMs from WT, *Hrh1* ^−/−^ *, and Hrh2* ^−/−^ mice were exposed to the fluorescently labeled *E. coli* (Figure 1b), or microspheres (Figure 1c) as discussed previously. *Hrh2* ^−/−^ cells, but not *Hrh1* ^−/−^ BMMs, yielded a defective phagocytosis phenotype when compared to WT cells from the same mouse strain. The histogram of FITC distribution in Figure 1b, left panel, demonstrated diminished phagocytosis of CFDA‐SE‐labeled *E. coli* in the *Hrh2* ^−/−^ cell population relative to the WT cell population. Due to variation in the amount of *E. coli* ingested by each cell, these peaks are broad, but clearly resolve into two cell populations. Median CFDA‐SE intensity is quantified for all experimental groups in Figure 1b, right panel. This phagocytic deficit is also found in cells exposed to Fluoresbrite YG microspheres. Microspheres provide the technical advantages of relatively uniform particle sizes lacking potentially confounding effects of living microbial cells as targets. In Figure 1c, left, distinctly microsphere‐negative and microsphere‐positive populations generated by WT and *Hrh2* ^−/−^ BMMs are shown. Signal peaks were created by increased fluorescence generated as fluorescent particles were ingested by phagocytes. Due to the discrete signals from the Fluoresbrite YG beads, the number of microsphere‐containing cells can be determined. We report the percentages of each macrophage population (WT, *Hrh1* ^−/−^, and *Hrh2* ^−/−^) containing microspheres in Figure 1c, right panel, showing defective phagocytosis in *Hrh2* ^−/−^ BMMs.

**Figure 2:**
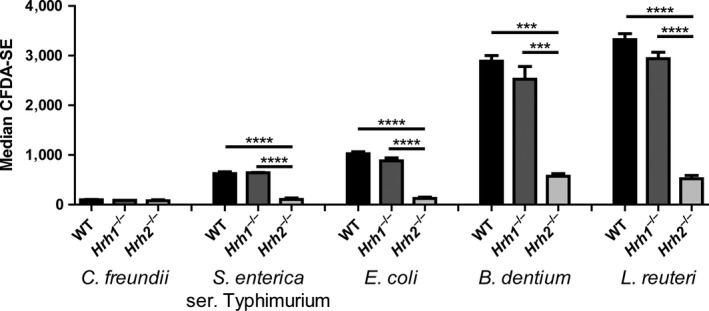
*Hrh2* ^−/−^ bone marrow‐derived macrophages are deficient in phagocytosis of phylogenetically diverse bacteria. To determine whether the lack of bacterial phagocytosis by *Hrh2* ^−/−^macrophages is specific to a certain group of bacteria, WT, *Hrh1* ^−/−^, and *Hrh2* ^−/−^ bone marrow‐derived macrophages were exposed to CFDA‐SE‐labeled *Citrobacter freundii*, *Salmonella enterica* ser. Typhimurium, and *Bifidobacterium dentium*, along with *Escherichia coli* and *Lactobacillus reuteri*, all at a MOI of 10. *Hrh2* ^−/−^ bone marrow‐derived macrophages exhibited a deficit in phagocytosis of all bacteria tested compared to WT and *Hrh1* ^−/−^ bone marrow‐derived macrophages, except for *C. freundii* which was not phagocytosed by WT or *Hrh1* ^−/−^ macrophages. Statistical analysis was performed by one‐way ANOVA of mean for each group. ***p < .001, ****p < .0001; *n* = 3

Along with *E. coli* and *L. reuteri*, murine BMMs were exposed to CFDA‐SE‐labeled *Citrobacter freundii*, *Salmonella enterica* serovar Typhimurium, and *Bifidobacterium dentium* (representing 4 different bacterial phyla) and phagocytosis was quantified by flow cytometry **(**Figure 2 **)**. *Citrobacter freundii* cells were not effectively phagocytosed by WT or histamine receptor‐deficient macrophages. *Hrh2* ^−/−^ BMMs, but not WT BMMs, were defective at phagocytosis of *S. enterica* serovar Typhimurium and *B. dentium*. To confirm that endogenous histamine was not playing a role in our model, we assessed BMM supernatants for histamine content by mass spectrometry. Histamine was not detected in any of the supernatants tested (Table A1 in Appendix), indicating that our findings regarding phagocytosis were not influenced by endogenous histamine produced by mouse cells. These data demonstrate that H2R is an important modulator of BMM‐mediated phagocytosis.

### 2.2 *Hrh2* ^−/−^ peritoneal macrophages are deficient in phagocytosis of fluorescent latex microspheres

**Figure 3:**
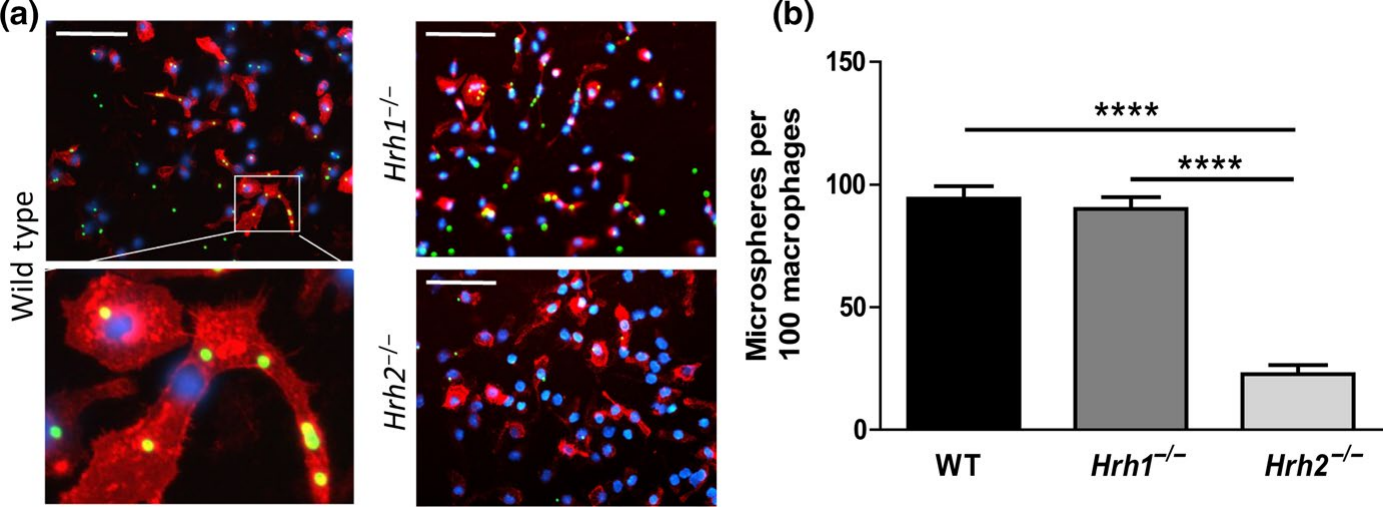
Phagocytosis is reduced in *Hrh2* ^−/−^ peritoneal macrophages. Phagocytosis of fluorescent microspheres was determined in WT, *Hrh1* ^−/−^, and *Hrh2* ^−/−^ peritoneal macrophages. (a) Fluorescence microscopy was used to analyze phagocytosis in WT, *Hrh1* ^−/−^, and *Hrh2* ^−/−^ peritoneal macrophages at 400x total magnification after 1‐hr exposure to Fluoresbrite YG microspheres 2 µm (green). Cells were stained with DAPI (blue) and TRITC‐conjugated anti‐mouse F4/80 antibody (red). Scale bars represent 50 µm length. Pop‐out of enlarged section showing WT macrophages associated with multiple beads. (b) Average number of microspheres associated with macrophages was calculated from counting microspheres and nuclei in 10 separate fields per genotype at 200x total magnification. Statistical analysis was performed by one‐way ANOVA of mean. ****p < .0001; *n* = 3

To study phagocytosis in peripheral macrophages that are more closely associated with the intestinal tract, peritoneal macrophages were isolated from the abdominal cavities of mice. To elicit enough cells for analysis, mice were injected intraperitoneally with Bio‐Gel P‐100, 4 days prior to macrophage isolation. We did not use thioglycollate in these experiments which is thought to interfere with subsequent phagocytosis analyses (Zhang, Goncalves, & Mosser, 2008). By exposing peritoneal macrophages with Fluoresbrite YG microspheres, we were able to validate decreased phagocytosis in *Hrh2* ^−/−^ in peritoneal macrophages and enumeration of clearly defined beads per macrophage. Isolated peritoneal macrophages were exposed to Fluoresbrite YG microspheres for 1 hr at a MOI of 10. Fluorescence microscopy of peritoneal macrophages reveals diminished phagocytosis of fluorescent microspheres by *Hrh2* ^−/−^ macrophages **(**Figure 3a). FIJI analysis demonstrates decreased numbers of microspheres (green foci) per one hundred (100) F4/80‐positive peritoneal macrophages (Figure 3b). The data support our in vitro findings with BMMs, indicating that H2R is a mediator of signaling pathways involved in phagocytosis by both bone marrow‐derived and peritoneal (peripheral) macrophages.

### 2.3 Expression of autophagy genes *Becn1* and *Atg12* is increased in *Hrh2* ^−/−^ macrophages

**Figure 4:**
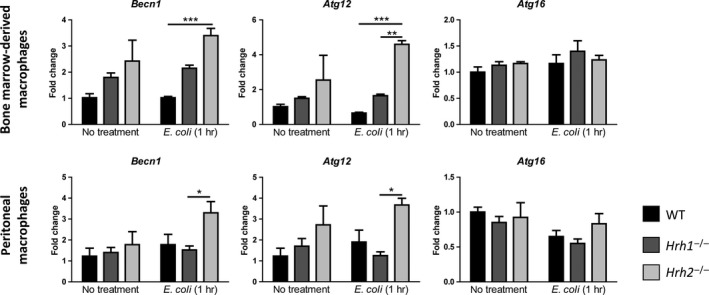
Expression of autophagy genes *Becn1* and *Atg12* is increased in *Hrh2* ^−/−^ macrophages. Expression of autophagy genes was analyzed in bone marrow‐derived macrophages and peritoneal macrophages by qPCR. WT, *Hrh1* ^−/−^, and *Hrh2* ^−/−^ macrophages were left untreated or exposed to *Escherichia coli* K‐12 at a MOI of 10 for 1 hr before lysis and RNA isolation. Autophagy gene expression was normalized to *Gapdh*. Statistical analysis was performed by two‐way ANOVA of mean with Bonferroni multiple comparisons. *p < .05, **p < .01, ***p < .001; *n* = 4

**Figure A2:**
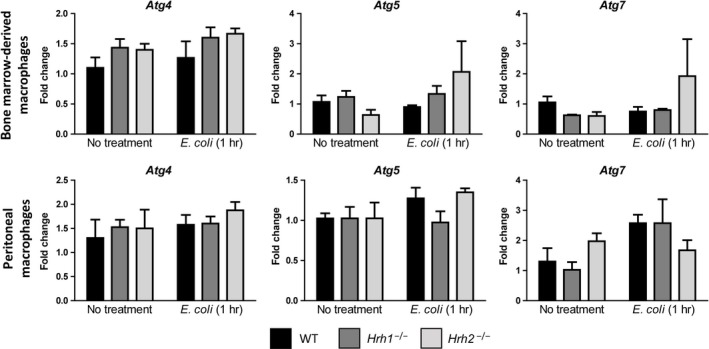
Expression of autophagy genes *Atg4*, *Atg5*, and *Atg7* is not different in histamine receptor‐deficient macrophages. Expression of autophagy genes was analyzed in bone marrow‐derived macrophages and peritoneal macrophages by qPCR. WT, *Hrh1* ^−/−^, and *Hrh2* ^−/−^ macrophages were left untreated or exposed to *Escherichia coli* K‐12 at a MOI of 10 for 1 hr before lysis and RNA isolation. Autophagy gene expression was normalized to *Gapdh*. Statistical analysis was performed by two‐way ANOVA of mean with Bonferroni multiple comparisons; *n* = 4

It was previously shown that enhancement of autophagy results in decreased phagocytosis by macrophages (Lima et al., 2011). Therefore, we sought to determine whether enhanced autophagy gene expression could be observed in *Hrh2* ^−/−^ macrophages. To do this, several key autophagy genes were quantified in bone marrow‐derived and peritoneal macrophages by qPCR following exposure to *E. coli* K‐12 (Figure 4 **)**. Of the genes analyzed, expression of *Becn1* and *Atg12*, which code for the autophagy pathway components Beclin‐1 and Autophagy‐related gene 12, was significantly increased in BMMs after 1‐hr exposure to *E. coli* K‐12 (Figure 4). Interestingly, we observed a trend toward greater expression of both genes in untreated *Hrh2* ^−/−^ BMMs and in peritoneal macrophages with and without exposure to *E. coli* (Figure 4). Expression of *Atg4*, *Atg5*, and *Atg7* genes was not different between WT and histamine receptor‐deficient macrophages (Figure A2 in Appendix).

### 2.4 Macrophage scavenger receptor 1 (MSR1) is reduced in *Hrh2* ^−/−^ macrophages

**Figure 5:**
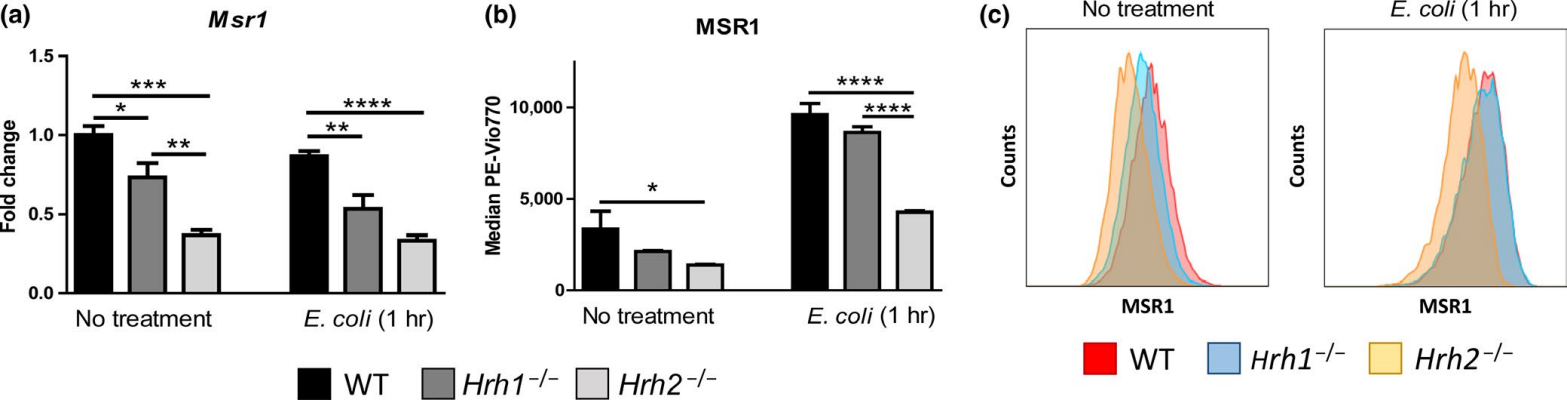
*Hrh2* ^−/−^ bone marrow‐derived macrophages exhibit decreased abundance of Macrophage Scavenger Receptor 1. MSR1 was analyzed by flow cytometry in untreated bone marrow‐derived macrophages and those exposed to *Escherichia coli* for 1 hr. Prior to analysis, macrophages were stained with a viability dye and then stained against the macrophage markers CD11b and F4/80 along with scavenger receptor directed antibodies. (a) Gene expression of *Msr1* was quantified in untreated and *E. coli*‐treated bone marrow‐derived macrophages by qPCR. Gene expression data were normalized to *Gapdh*. (b) Median fluorescence intensity was determined in the PE‐Vio770 channel, and MSR1 surface abundance was compared. (c) Representative flow cytometry histograms of PE‐Vio770 intensity in WT, *Hrh1* ^−/−^, *Hrh2* ^−/−^ populations of bone marrow‐derived macrophages demonstrate a leftward peak shift in *Hrh2* ^−/−^ populations compared to WT and *Hrh1* ^−/−^ populations. Statistical significance was determined by two‐way ANOVA of mean with Bonferroni multiple comparisons. *p < .05, **p < .01, ***p < .001, ****p < .0001; *n* = 4

Loss of the autophagy‐related gene *Atg7* in murine macrophages has been demonstrated to increase phagocytosis due to increased scavenger receptor production (Bonilla et al., 2013). To determine whether scavenger receptors were involved in our phenotype, we examined expression of key scavenger receptor genes in WT, *Hrh1* ^−/−^, and *Hrh2* ^−/−^ macrophages exposed to *E. coli* for 1 hr (MOI 10). Reduced expression of scavenger receptor gene *Msr1* was observed in *Hrh2* ^−/−^ macrophages (Figure 5a). Consistent with qPCR data, flow cytometric analysis yielded decreased cell surface abundance of MSR1 in *Hrh2* ^−/−^ BMMs (Figure 5b,c). This finding was reflected in the average of median surface fluorescence intensities of MSR1 and representative flow cytometry histograms. The *Hrh2* ^−/−^ population (orange peak) was shifted to the left compared to WT (red peak) and *Hrh1* ^−/−^ (blue peak) indicating lesser abundance of MSR1. The data demonstrate transcriptional downregulation of MSR1 production which may explain, in part, the defective phagocytosis of Hrh2‐deficient macrophages (Bonilla et al., 2013).

### 2.5 *Hrh1* and *Hrh2* do not influence TNF production or chemotaxis in bone marrow macrophages

**Figure A3:**
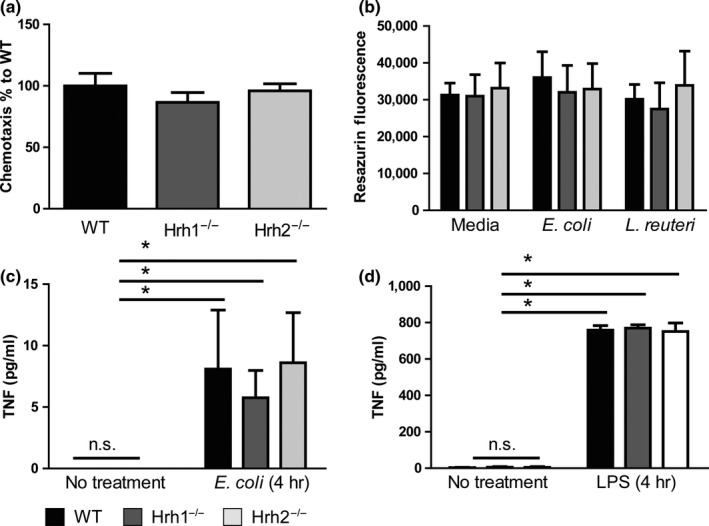
Histamine receptor status does not affect chemotaxis or TNF production in bone marrow‐derived macrophages. Cell viability and key macrophage functions were quantified in WT and histamine receptor‐deficient BMMs. (a) Chemotaxis is not different in histamine receptor‐deficient macrophages, and analysis of viability (b) shows that exposure to bacteria for 1 hr does not modulate viability in histamine receptor‐deficient macrophages. TNF production was quantified in WT and histamine receptor‐deficient macrophages after exposure to *Escherichia coli* for 4 hr (c) or LPS alone for 4 hr (d). Both treatments induced TNF production, but not differently in histamine receptor‐deficient macrophages. Statistical significance was determined by two‐way ANOVA of mean with Bonferroni multiple comparisons. *p < .05

Conflicting evidence exists regarding the role of histamine receptors in macrophage chemotaxis (Czerner et al., 2014; Radermecker et al., 1989). We observed significant changes in phagocytosis in response to histamine receptor status, so we next addressed whether our macrophages were capable of chemotaxing toward *E. coli* and responding to *E. coli* or LPS via cytokine production. Using fluorescently tagged macrophages and chemotaxis chambers (8 µm pore size), we observed no difference in chemotaxis of our macrophages toward live *E. coli* (Figure A3 in Appendix). Since metabolic stress has been shown to modulate macrophage chemotaxis (Qiao et al., 2009), we examined metabolic activity of our macrophages using the compound resazurin. No differences were found between genotypes of bacterial treatment (*E. coli* or *L. reuteri*) (Figure A3 in Appendix). To determine whether any changes existed in baseline cytokine production, bone marrow macrophages were exposed to 4 hr of *E. coli* K12 (MOI of 10) or 1 µg/ml LPS and secreted TNF was examined by ELISA (Figure A3 in Appendix). Compared to treatment naïve macrophages, addition of *E. coli* or LPS induced TNF secretion in WT bone marrow macrophages. Interestingly, no differences were observed between Hrh1^−/−^ and Hrh2^−/−^ and control WT bone marrow macrophages, indicating that histamine receptor status does not influence TNF production. These data suggest that phagocytosis defects are not due to chemotaxis or the TLR activation in Hrh2^−/−^ macrophages, but likely due to changes in scavenger receptor abundance. Collectively, these data suggest that Hrh2 influences autophagy pathways and scavenger receptors thereby modulating phagocytosis.

## 3 DISCUSSION

**Figure 6:**
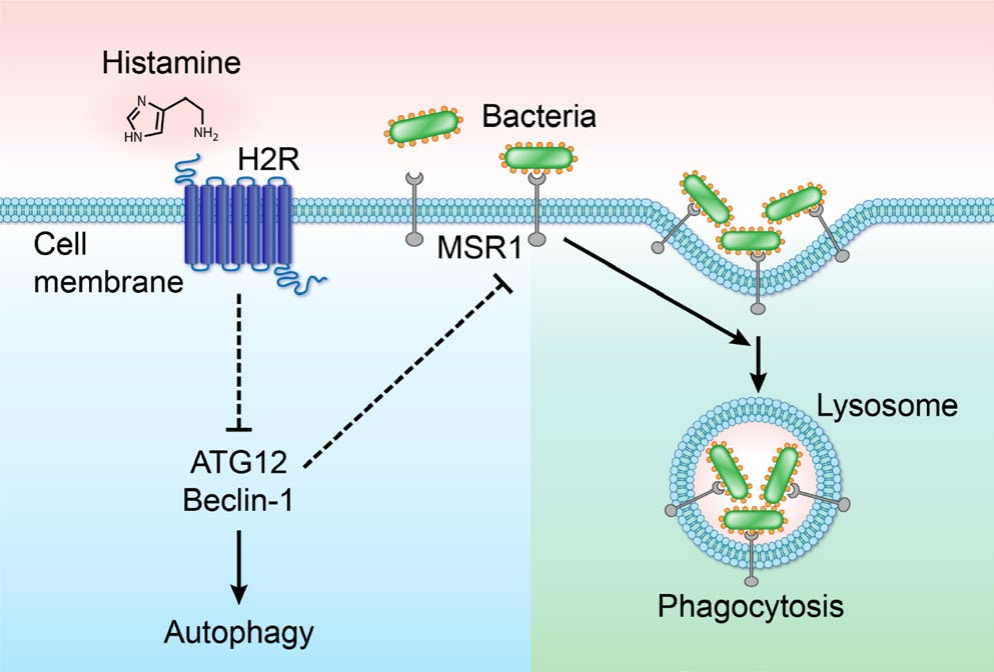
Schematic representation of the role of Hrh2 in macrophage‐mediated phagocytosis. Histamine H2 receptor signaling is required for appropriate MSR1 production and efficient phagocytosis by suppression of autophagy genes *Atg12* and *Becn1* in macrophages. Deletion of *Hrh2* results in increased *Atg12* and *Becn1* expression and decreased Macrophage Scavenger Receptor 1 cell surface abundance and reduced phagocytosis

Previous studies have demonstrated possible connections between histamine signaling and phagocytosis, but the roles of specific histamine receptors in innate immunity and phagocytosis remain unclear. Herein, we identify defects in phagocytosis in mammalian macrophages lacking the histamine receptor *Hrh2* ^−/−^ but such defects in phagocytosis were not apparent in macrophages lacking the histamine receptor *Hrh1* ^−/−^. Our fluorescence microscopy revealed observable decreases in the phagocytosis of fluorescent bacteria and microparticles by *Hrh2* ^−/−^ bone marrow‐derived and peritoneal macrophages. These findings were confirmed by flow cytometry. Additionally, we demonstrate increased expression of autophagy‐related genes *Atg12* and *Becn1*. Finally, we found that *Hrh2* ^−/−^ macrophages exhibited diminished gene expression and cell surface abundance of the scavenger protein MSR1. Based on these findings, we conclude that signaling via H2R is involved in regulation of autophagy and scavenger receptor gene expression, thereby influencing macrophage function. Interestingly, we found that defects in phagocytosis were not related to chemotaxis or TLR activation, as *Hrh2* ^−/−^ BMMs exhibited no defects in chemotaxis toward *E. coli* or production of TNF. Taken together, we propose the model illustrated in Figure 6 to describe the processes by which H2R is involved in phagocytosis.

The relative capacity for autophagy was determined by quantifying autophagy gene expression in both BMMs and peritoneal macrophages. *Becn1* and *Atg12* were both significantly increased in expression after exposure to *E. coli* in *Hrh2* ^−/−^ BMMs. The data suggest that increased autophagy may result from a loss of H2R signaling. Scavenger receptor transcription has been linked to autophagy proteins previously (Bonilla et al., 2013). The inverse correlation of autophagy and phagocytosis is the result of competition for cellular resources by the two pathways, autophagy and phagocytosis. It has been shown that knocking out a key autophagy gene in mice, *Atg7*, results in increase phagocytosis by macrophages along with increased scavenger receptor expression (Bonilla et al., 2013). Alternatively, inducing autophagy leads to reduced phagocytosis (Thomas et al., 2012). In our model, we see that decreased phagocytosis in *Hrh2* ^−/−^ macrophages was likewise accompanied by an increase in expression of two key autophagy genes, *Atg12* and *Becn1*. Formation of both phago‐lysosomes and auto‐lysosomes requires contribution of membrane material from the plasma membrane; thus, it has been hypothesized that limited cellular membrane resources is the underlying cause of the apparent negative feedback control between autophagy and phagocytosis (Thomas et al., 2012). Bonilla *et al*. demonstrate the molecular mechanism responsible for the increase in scavenger receptor abundance in *Atg7* ^−/−^ macrophages. This group demonstrates that increased *Marco* and *Msr1* expression was caused by increased activity of nuclear factor (erythroid‐derived 2)‐like 2 (NFE2L2), the transcription factor for these scavenger receptors. In autophagy‐impaired macrophages, accumulation of p62, which is embedded in the autophagosome, is more available for activation of NFE2L2; thus, *Marco* and *Msr1* expression is increased (Bonilla et al., 2013). Accordingly, in cells with highly active autophagy, a lower abundance of free p62 may result in less activation of NFE2L2 and diminished scavenger receptor transcription. In *Hrh2* ^−/−^ macrophages, we did not find changes in *Atg7* expression, or in expression of *Atg4*, *Atg5*, or *Atg16* (Figure A2 in Appendix) which along with *Becn1* and *Atg12* have been studied in one or more aspects of immunological regulation (Virgin & Levine, 2009). The data suggest, however, that the mechanistic negative feedback of phagocytosis by autophagy extends to other autophagy‐related gene products as well, as we detected increased *Becn1* and *Atg12* expression accompanied by decreased *Msr1* expression and MSR1 surface abundance.

**Figure A4:**
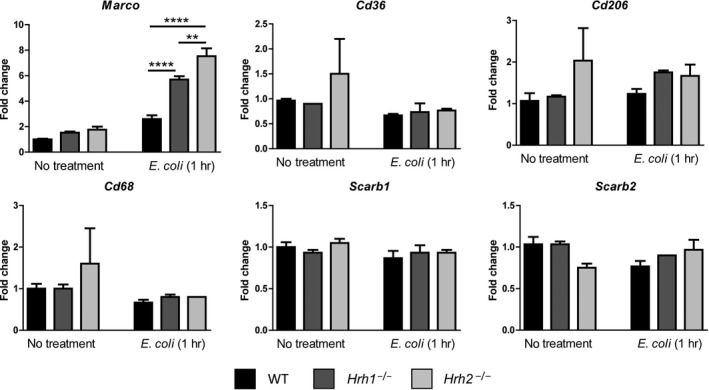
Scavenger receptor gene expression analysis. Expression of scavenger receptor genes was analyzed by qPCR. WT, *Hrh1* ^−/−^, and *Hrh2* ^−/−^ bone marrow‐derived macrophages were left untreated or exposed to *Escherichia coli* K‐12 at a MOI of 10 for 1 hr before lysis and RNA isolation. Scavenger receptor gene expression was normalized to *Gapdh*. Statistical analysis: Two‐way ANOVA of mean with Bonferroni multiple comparisons. **p < .001, ****p < .0001; *n* = 4

**Figure A5:**
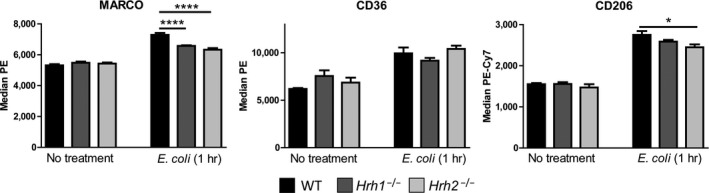
Scavenger receptor protein analysis. Scavenger receptors were analyzed by flow cytometry in untreated bone marrow‐derived macrophages and those exposed to *Escherichia coli* for 1 hr. Prior to analysis, macrophages were stained with a viability dye and then stained against the macrophage markers CD11b and F4/80 along with scavenger receptor directed antibodies. Median fluorescence intensity was determined in the PE and PE‐Vio770 channels, and protein surface abundance was compared. Statistical significance was determined by two‐way ANOVA of mean with Bonferroni multiple comparisons. *p < .01, ****p < .0001; *n* = 4

Scavenger receptors are transmembrane glycoproteins that include CD36, CD68, SR class A, and SR class B receptors, and binding of receptor to their ligands mediates uptake and clearance. The receptors mediate multiple physiologically important cell processes such as the uptake of oxidized lipoproteins as well as recognition and initiation of phagocytosis of microbes by macrophages (Rich et al., 2013). MSR1 is a class A scavenger receptor that is present on macrophages and exhibits broad ligand specificity (Areschoug & Gordon, 2009). *Msr1* is upregulated on macrophages in response to exposure to bacteria and results in binding and removal of bacteria by phagocytosis (Arredouani et al., 2005). We examined the expression of key scavenger receptors by qPCR and confirmed the receptor patterns by flow cytometry when possible. *Msr1*, *Marco*, *Cd36*, *Cd206*, *Cd68*, *Scarb1*, and *Scarb2* expression was quantified with and without exposure to *E. coli* (Figure A4 in Appendix). MSR1, MARCO, CD36, and CD206 surface abundance was quantified by flow cytometry as well (Figure A5 in Appendix). Of the examined receptors, *Msr1* gene expression and MSR1 surface abundance were markedly decreased in *Hrh2* ^−/−^ BMMs.

Collectively, the work presented herein demonstrates that H2R influences both autophagy and scavenger receptors to enable phagocytosis in macrophages. To complete the mechanistic understanding of this model, future experiments should be focused on determining if NFE2L2 activation is reduced in *Hrh2* ^−/−^ macrophages and how this mechanism may be regulated by H2R signaling. Aside from understanding the role of H2R signaling in macrophage phagocytosis, future work should be done to understand how inhibition of H2R with pharmaceutical antagonists may be affecting infection in humans. H2R blockers are prescribed and are available for over‐the‐counter purchase for treatment of esophageal reflux, and there is a correlation between use of H2R blockers and increased risk of some infections in humans (Cohen, Bueno de Mesquita, & Mimouni, 2015). Therefore, work should be done to determine whether H2R blocker use in humans, especially immunocompromised individuals, impairs bacterial clearance by reducing macrophage phagocytic ability.

## 4 MATERIALS AND METHODS

### 4.1 Histamine receptor‐deficient mouse models

*Hrh1* and *Hrh2* receptor‐deficient mouse models on the C57BL/6J background were generated by the Genetically Modified Mouse Core at Baylor College of Medicine using CRISPR‐Cas9‐mediated deletion of functional gene regions. Mice were bred and housed under specific pathogen‐free (SPF) conditions with a 12‐hr light cycle, at the Feigin Tower vivarium at Texas Children's Hospital, Houston, TX. For genotyping, tail clippings were digested with proteinase K using DirectPCR Lysis Reagent (Viagen, 101‐T) and incubated overnight at 55ºC. All digests were terminated by incubation at 80ºC for 45 min. Crude lysates were used with genotyping primers (Table A2 in Appendix) and NEB Taq DNA polymerase (New England Biolabs, M0270L) for standard PCR analysis. PCR products were run on 1.5% sodium borate agarose gels and visualized using EZ vision3 dye (VWR, N472). Using Primer Set 1, wild‐type (WT) mice generate a 530 bp product, while *Hrh1*‐deficient mice generated a 390 bp product. Using Primer Set 2, WT mice generated a 627 bp product while *Hrh2*‐deficient mice generated a 310 bp product. Mice were allocated to experimental groups by genotype, and no randomization or blinding was used for experiments. BMMs and peritoneal macrophages were harvested from these mice after euthanasia.

### 4.2 Bacteria strains and culture conditions

*Escherichia coli* K‐12 (Carolina Biologicals) was cultured in Luria–Bertani (LB) bacteriological media (Invitrogen), and *C. freundii* and *S. enterica* serovar Typhimurium were cultured in brain heart infusion (BHI) bacteriological media (Becton Dickinson) for 24 hr, aerobically at 37°C while shaking. *Lactobacillus reuteri* ATCC PTA 6475 (BioGaia AB) and *B. dentium* ATCC 27678 (ATCC) were cultured in De Man, Rogosa and Sharpe (MRS) bacteriological media (Becton Dickinson) for 24 hr at 37°C, in an Anaerobe Systems AS‐580 anaerobic chamber. In preparation for fluorescent labeling, bacteria were washed twice with sterile phosphate‐buffered saline without calcium and magnesium (PBS; ThermoFisher, 14190144) by centrifugation at 4,000 g for 10 min at room temperature. The bacteria were then resuspended in PBS containing 1 mM glucose and 50 µM 5‐(and‐6)‐carboxyfluorescein diacetate, succinimidyl esters (CFDA‐SE) (Life technologies, C1157). The bacteria were mixed by vortexing and incubated at 37°C in the dark for 1 hr. After incubating with CFDA‐SE, the bacteria were washed in PBS. The concentrations of bacteria were estimated by measuring optical density at 600 nm (OD_600_) using the Bio‐Rad UV/visible SmartSpec Plus spectrophotometer.

### 4.3 Macrophage models

To examine phagocytosis in mouse macrophages, bone marrow‐derived macrophages were generated from adult WT and histamine receptor‐deficient C57BL/6J mice by culturing bone marrow cells as previously described (Trouplin et al., 2013). Briefly, bone marrow cells were harvested by flushing tibias and femurs, in the presence of 50 nM recombinant murine macrophage colony‐stimulating factor (M‐CSF) (PeproTech, 315–02) in complete DMEM (DMEM media (ATCC, 30‐2002) with heat‐inactivated fetal bovine serum (FBS) (Thermo Fisher, 10438026), and 1 × antibiotic/antimycotic (Thermo Fisher, 15240062)) for seven days. Peritoneal macrophages were elicited from adult mice by intraperitoneal injection of 2% Bio‐Gel P‐100 (Bio‐Rad, 1504174), followed by a 4‐day rest period as previously described (Ray & Dittel, 2010). After the rest period, macrophages were harvested by peritoneal lavage with 10 ml prewarmed PBS. The resulting peritoneal cell suspension was washed by centrifugation at 600 g for 10 min at 4˚C and then resuspended in complete DMEM and allowed to attach to nontissue culture treated, plastic Petri dishes for 6 hr before washing twice with PBS and detaching with 0.25% Trypsin‐EDTA (Thermo Fisher, 25200‐056).

### 4.4 Flow cytometry

For flow cytometric analysis of phagocytosis, BMMs were exposed to fluorescently labeled bacteria or 2 µm Fluoresbrite YG microspheres (Polysciences, Inc., 18338‐5) at a MOI of 10 for 1 hr and then washed with PBS and stained with fixable viability dye eFluor 660 (Thermo Fisher, 65‐0864‐14) for 30 min, in the dark, on ice. The BMMs were then washed with PBS and fixed in 1% paraformaldehyde before analysis. Surface abundance of MSR1 was evaluated with a flow cytometry validated antibodies for MSR1 (clone REA148, PE‐Vio770 conjugated, Miltenyi Biotec 130‐105‐522 CD36 (clone HM36, PE conjugated, BioLegend 102606), MARCO (clone ED31, PE conjugated, Bio‐Rad MCA1849PE), and CD206 (clone MR6F3, PE‐Cy7 conjugated, ThermoFisher 25‐2061‐80). For surface marker analysis, BMMs were exposed to *E. coli* K‐12 at a MOI of 10 in 6‐well plates and then washed with PBS and detached by trypsinization. The macrophages were then stained with fixable viability dye eFluor 660, and F_c_ receptors were blocked with anti‐CD16/32 antibody (Clone 93) from ThermoFisher. BMMs were stained with each scavenger receptor antibody for 30 min on ice and then washed and fixed in 1% paraformaldehyde and analyzed. Cells were analyzed on a FACSCanto (BD Bioscience) using FACSDiva software, and data were analyzed using FlowJo V10 (Tree Star).

### 4.5 Fluorescence microscopy

For microscopic evaluation, macrophages were stained with trypan blue and counted with the Invitrogen Countess II automated cell counter. The cells were then seeded at a density of 3 × 10^5^ live cells per chamber in 4‐chamber glass slides (Thermo Fisher, 154526) and allowed to attach for 12 hr. Complete media was replaced with prewarmed serum‐free media for 30 min, and then, CFDA‐SE‐labeled bacteria or 2 µm Fluoresbrite YG microspheres were added, at a MOI of 10 for 1 hr. Macrophages were then washed and fixed for staining with 4% paraformaldehyde (PFA) for 30 min at room temperature. BMMs were permeabilized with 0.1% Triton X‐100 for 30 min at room temperature and stained with 100 nM Acti‐stain 555 Phalloidin (Cytoskeleton Inc., PHDH1‐A) for 30 min in the dark at room temperature to visualize the actin cytoskeleton. Additionally, peritoneal macrophages were stained with an anti‐F4/80 antibody (F4/80 Monoclonal Antibody (Clone A3‐1) macrophage marker, Life Technologies, MA5‐16624) overnight at 4ºC. Following immunostaining, nuclei were visualized with 10 µg/ml Hoechst 33342 and cover slipped with Fluoromount Aqueous Mounting Medium (Sigma‐Aldrich F4680).

Immunostaining was examined on an upright wide‐field epifluorescence Nikon Eclipse 90i (Nikon) with a 10 × ocular lens and the following objectives: 20 × Plan Apo (NA 0.75) differential interference contrast (DIC) objective and a 40 × Plan Apo (NA 0.95) DIC. All images were recorded using a CoolSNAP HQ2 camera (Photometrics) with a SPECTRA X LED light source (Lumencor). FIJI (Fiji Is Just ImageJ) software was used to perform semiquantitative analysis of fluorescent stains by tabulating mean pixel intensity (National Institutes of Health) in ten regions/ per chamber, *n* = 4–10 mice/group.

### 4.6 Quantitative real‐time PCR

To quantify expression of autophagy and scavenger receptor genes (*Becn1*, *Atg4*, *Atg5*, *Atg7*, *Atg12*, and *Atg16*), RNA was isolated from BMMs and peritoneal macrophages after treatment with *E. coli* K‐12. RNA was isolated from TRIZOL treated cell lysate samples using a column purification kit (QIAGEN, 217004) according to the manufacturer's instructions. Complementary DNA was prepared using the SensiFAST cDNA synthesis kit (Bioline, BIO‐65054). Quantitative PCR analysis was then performed using Fast SYBR Green (Applied Biosystems 4385618) and amplified on the Applied Biosystems QuantStudio3 instrument. *Gapdh* was used as the house keeping gene, and relative gene expression was analyzed using the comparative Ct method (2‐∆∆Ct method). Primers for qPCR analysis are listed in Table A3 in Appendix.

### 4.7 Liquid chromatography/ mass spectrometry (LC‐MS)

To examine endogenous production of histamine by macrophages, overnight supernatant was collected from macrophages. To prepare samples for LC‐MS analysis, 30 µl of each sample was dried in a SpeedVac for 5 hr. Once dried, 30 µl of histamine‐d4 solution was added to each sample and vortexed for 1 min. Samples were then loaded into 0.5 ml autosampler vials for quantification. The histamine‐d4 internal standard solution was prepared at a concentration of 20 ng/ml of histamine‐d4 in water with 0.1% FA.

Chromatography was performed on a Shimadzu Nexera‐XR HPLC system consisting of an SIL‐20ACxr autosampler, a CTO‐20AC column over and 2 LC‐20ADxr binary pumps. Samples (5 µl) were loaded onto a Phenomenex (Torrance, CA) 1 mm × 50 mm phenylhexyl reversed phased column equipped with a Phenomenex phenylhexyl 4 mm × 2 mm guard column. The aqueous mobile phase (A) consisted of H_2_O:ACN:FA:PFHA (99.3:0.5:0.1:0.1 v/v/v/v), and the organic mobile phase (B) consisted of H_2_O:FA (99.9:0.1 v/v). Column flow was 80 µl/min, and 5 µl of sample was injected onto the column and eluted with a constant mobile phase flow rate of 80 µl/min. The elution gradient was optimized as follows: started from 10% B and increased to 70% B over 5 min; ramp to 80% B for 6 s and held for 1 min; ramp back to 10% B over 6 s and maintained at 10% for a total chromatographic run time of 12 min to re‐equilibrate.

Selected reaction monitoring was performed on a Sciex (Framingham, MA) 6500 QTRAP with a Turbo V source. The mass spectrometer was operated in the positive ion mode under the following conditions: curtain gas: 20 psi; collision gas: HIGH; spray voltage: 4.5 kV; ion source gas 1:10 psi; ion source gas 2:2 psi; interface heater temperature: 175°C; Q1 and Q3 resolution: unit; scan time: 100 mS; de‐clustering potential: 100 V; entrance potential: 8 V; and collision exit potential: 10 V. The instrument was calibrated by using Sciex PPG calibration standard and tuned to the manufacturer's specifications. SRM transitions monitored for histamine were 112 → 95 (20eV) and 112 → 68 (30eV). For histamine‐d4, the SRM transitions 116 → 99 (20eV) and 116 → 72 (30eV) were monitored. Data were acquired with Analyst® Software (ver 1.6.2), and quantification performed using Multiquant™ Software (ver 3.0.1).

### 4.8 Chemotaxis and viability assays and cytokine quantification by ELISA

For analysis of chemotaxis, macrophages were fluorescently tagged with 10 µM CFDA‐SE for 15 min at 37ºC, 5% CO_2_, similar to CFDA‐SE staining for bacteria. Fluorescence was confirmed by microscopy. Cell number and viability was assessed by trypan blue staining using the Countess II as described previously. Cells were resuspended at 4 × 10^5^ cells per well in chemotaxis medium (RPMI 1640 supplemented with 0.1% bovine serum albumin (Sigma‐Aldrich, A7030). Macrophages were placed in Corning HTS 8 µm Transwell 96 well permeable supports (Sigma, CLS3374). Chemotaxis medium containing nontagged *E. coli* K12 (MOI = 10) was added to the bottom wells of the chamber, and the membrane was inserted. The chamber was incubated for 4 hr at 37ºC, 5% CO_2_, and 95% humidity to allow for chemotaxis. Chemotaxis was quantified by measuring the fluorescence of macrophages that had traversed to the bottom chamber (excitation: 485 nm, emission: 528 nm).

To assess the metabolic activity of our macrophages, the resazurin assay was implemented as previously described (Feng & Cohen, 2013). Briefly, the dye resazurin (7‐hydroxy‐3H‐phenoxazin‐3‐one 10‐oxide) (Sigma‐Aldrich, R7017) was added at a final concentration of 44 µM, and plates were incubated for 3 hr at 37°C, 5% CO_2_. Cell viability was measured by reading the fluorescence resulting from resazurin reduction to resorufin using a microplate spectrofluorometer at an excitation wavelength of 570 nm and an emission wavelength of 600 nm.

Quantitative ELISA was used to determine TNF concentrations in cell‐free supernatants (Affymetrix eBioscience 88‐7324‐22). Each condition was run in biological triplicate and in technical duplicate. Prior to ELISA, protein concentrations were measured and normalized for each group according to the manufacturer's instructions (R&D Systems). Colorimetric absorbances were measured spectrophotometrically, and TNF concentrations were calculated from the standard curves according to manufacturer's instructions.

### 4.9 Statistical analysis

All sample sizes (*n*‐values) indicated in each figure legend correspond to independent biological replicates. Data presented in graphs represent averages and standard deviations. Comparisons between groups were made with either one‐ or two‐way ANOVA (GraphPad v5.04). A *p*‐value of .05 or less was considered significant.

## CONFLICT OF INTERESTS

J. Versalovic serves on the scientific advisory boards of Seed Health, Biomica, and Plexus Worldwide. J. Versalovic receives unrestricted research support from Biogaia AB.

## AUTHOR CONTRIBUTIONS

R.F., M.A.E., Y.M.‐A., and J.V. conceived and designed research; R.F., M.A.E., Z.S., A.Hall, B.H., B.P.G., A.M., and A.Haag performed experiments; R.F., M.A.E., A.Haag, Y.M.‐A., and J.V analyzed data; R.F., Y.M.‐A., and J.V. interpreted results of experiments; R.F. prepared figures; R.F. drafted manuscript; R.F., M.A.E., Z.S., A.Hall, Y.M.‐A., and J.V. edited and revised manuscript; and R.F., Y.M.‐A., and J.V. approved final version of manuscript.

## ETHICS STATEMENT

All experiments were performed with the approval of the Institutional Animal Care and Usage Committee of Baylor College of Medicine under protocol number AN‐6109.

## Data Availability

All primer sequence data are provided in Tables A2 and A3 in Appendix.

## APPENDIX 1

**Table A1:** WT and histamine receptor‐deficient mouse macrophages do not excrete measurable amounts of histamine

	Histamine concentration	
	BMM culture media	111 ng/ml control
Wild type	Not detected	85.3 ± 2.7 ng/ml
*Hrh1* ^−/−^	Not detected	77.6 ± 6.8 ng/ml
*Hrh2* ^−/−^	Not detected	87.4 ± 3.2 ng/ml

Note

Mass spectrometry analysis of the histamine content of DMEM cell culture from bone marrow‐derived macrophage culture or fresh DMEM + 111 ng/ml histamine. *N* = 3

**Table A2:** Histamine receptor‐deficient mouse genotyping primers

Primer	Sequence (5’−3’)
*Hrh1* P1	GTCACAGAGGGAGCATGCTT
*Hrh1* P2	CTGTCCTGTTCCCCTCACAC
*Hrh1* P3	GCTCTGCTTTCTACCCAGGG
*Hrh2* P1	CAGACACCCTGCAGACTGAG
*Hrh2* P2	TCCTCATGGAGACTGAGGCA
*Hrh2* P3	AAGAAGGGCCTCAGAGCTCT

Note

Primer sets used to evaluate *Hrh1* and *Hrh2* gene regions in histamine receptor knockout mouse lines.

**Table A3:** Quantitative real‐time PCR primers

Primer	Sequence
*Atg4* Forward	GGGAACTGGCCCTACTTCAGA
*Atg4* Reverse	TCCACCTCCAATCTCGACCTA
*Atg5* Forward	GGACAGCTGCACACACTTGG
*Atg5* Reverse	TGGCTCTATCCCGTGAATCAT
*Atg7* Forward	GGCCTTTGAGGAATTTTTTGG
*Atg7* Reverse	ACGTCTCTAGCTCCCTGCATG
*Atg12* Forward	TGAATCAGTCCTTTGCCCCT
*Atg12* Reverse	CATGCCTGGGATTTGCAGT
*Atg16* Forward	GCCCAGTTGAGGATCAAACAC
*Atg16* Reverse	CTGCTGCATTTGGTTGTTCAG
*Becn1* Forward	GGCCAATAAGATGGGTCTGA
*Becn1* Reverse	GCTGCACACAGTCCAGAAAA
*Cd36 Forward*	ATGGGCTGTGATCGGAACTG
*Cd36 Reverse*	TTTGCCACGTCATCTGGGTTT
*Cd68* Forward	TGTCTGATCTTGCTAGGACCG
*Cd68* Reverse	GAGAGTAACGGCCTTTTTGTGA
*Cd206* Forward	ATGCTGTAGTACCGGAGGGT
*Cd206* Reverse	CATCACTCCAGGTGAACCCC
*Gapdh* Forward	TGCACCACCAACTGCTTAGC
*Gapdh* Reverse	GGCATGGACTGTGGTCATGAG
*Msr1* Forward	ACTCCCTGATCATATTGGGGC
*Msr1* Reverse	GGTAAAAGGTGCCAGGGGAT
*Scarb1* Forward	TTTGGAGTGGTAGTAAAAAGGGC
*Scarb1* Reverse	TGACATCAGGGACTCAGAGTAG
*Scarb2* Forward	AGAAGGCGGTAGACCAGAC
*Scarb2* Reverse	CTGTAGGTGTATGGCCCCAC

Note

Primer pairs used to evaluate expression of autophagy and scavenger receptor genes.
